# Saxagliptin added to a submaximal dose of sulphonylurea improves glycaemic control compared with uptitration of sulphonylurea in patients with type 2 diabetes: a randomised controlled trial

**DOI:** 10.1111/j.1742-1241.2009.02143.x

**Published:** 2009-09

**Authors:** A R Chacra, G H Tan, A Apanovitch, S Ravichandran, J List, R Chen, for the CV181-040 Investigators

**Affiliations:** 1 Diabetes Center, Federal University of São PauloSão Paulo, Brazil; 2 Cebu Doctors’ University College of Medicine, Cebu Doctors’ University HospitalCebu City, Philippines; 3 Bristol-Myers SquibbPrinceton, NJ, USA

## Abstract

**Aims:**

Assess the efficacy and safety of saxagliptin added to a submaximal sulphonylurea dose vs. uptitration of sulphonylurea monotherapy in patients with type 2 diabetes and inadequate glycaemic control with sulphonylurea monotherapy.

**Methods and patients:**

A total of 768 patients (18–77 years; HbA_1c_ screening ≥ 7.5 to ≤ 10.0%) were randomised and treated with saxagliptin 2.5 or 5 mg in combination with glyburide 7.5 mg vs. glyburide 10 mg for 24 weeks. Blinded uptitration glyburide was allowed in the glyburide-only arm to a maximum total daily dose of 15 mg. Efficacy analyses were performed using ANCOVA and last-observation-carried-forward methodology.

**Results:**

At week 24, 92% of glyburide-only patients were uptitrated to a total glyburide dose of 15 mg/day. Saxagliptin 2.5 and 5 mg provided statistically significant adjusted mean decreases from baseline to week 24 vs. uptitrated glyburide, respectively, in HbA_1c_ (−0.54%, −0.64% vs. +0.08%; both p < 0.0001) and fasting plasma glucose (−7, −10 vs. +1 mg/dl; p = 0.0218 and p = 0.002). The proportion of patients achieving an HbA_1c_ < 7% was greater for saxagliptin 2.5 and 5 mg vs. uptitrated glyburide (22.4% and 22.8% vs. 9.1%; both p *<* 0.0001). Postprandial glucose area under the curve was reduced for saxagliptin 2.5 and 5 mg vs. uptitrated glyburide (−4296 and −5000 vs. +1196 mg·min/dl; both p < 0.0001). Adverse event occurrence was similar across all groups. Reported hypoglycaemic events were not statistically significantly different for saxagliptin 2.5 (13.3%) and 5 mg (14.6%) vs. uptitrated glyburide (10.1%).

**Conclusion:**

Saxagliptin added to submaximal glyburide therapy led to statistically significant improvements vs. uptitration of glyburide alone across key glycaemic parameters and was generally well tolerated.

**What’s known**

- Initial antihyperglycaemic monotherapy is frequently insufficient to enable patients with type 2 diabetes to achieve or sustain glycaemic targets.
- Sulphonylurea therapy has demonstrated efficacy in improving glycaemic control; however, it is also associated with limitations, including the potential for weight gain and an increased risk of hypoglycaemia with sulphonylurea-induced hyperinsulinaemia.

**What’s new**

- The availability of newer agents such as dipeptidyl peptidase-4 inhibitors may allow for additional therapeutic combinations to improve glycaemic control without significantly increasing the risk of adverse effects.
- This study evaluated the safety and efficacy of saxagliptin added to submaximal-dose sulphonylurea (glyburide) therapy, compared with uptitration of sulphonylurea (glyburide) monotherapy for 24 weeks in patients with inadequate glycaemic control with submaximal-dose sulphonylurea therapy alone.

## Introduction

As a result of the progressive decline in functional insulin-producing beta cells, the majority of patients with type 2 diabetes eventually require combination drug therapy to achieve and maintain glycaemic targets (1–3). The availability of newer agents may allow for different therapeutic combinations to improve glycaemic control without increasing the risk of adverse effects (4,5).

Saxagliptin is a potent, selective dipeptidyl peptidase-4 (DPP-4) inhibitor, specifically designed for extended inhibition of the DPP-4 enzyme (6,7). DPP-4 inhibitors enhance the levels of the glucoregulatory hormones glucagon-like peptide-1 (GLP-1) and glucose-dependent insulinotropic peptide (GIP), thereby acting to promote insulin synthesis and release, and suppress glucagon secretion, among other important glucoregulatory effects (8). DPP-4 inhibitors are associated with a favourable safety profile, including a low risk of hypoglycaemia because of the glucose-dependent nature of incretin hormone activity, a neutral effect on body weight and the potential for improved beta-cell function (8,9). Proof of concept for saxagliptin was previously established in a 12-week dose-ranging trial (dose range: 2.5–40 mg) (6,7). In a phase 3 clinical trial, saxagliptin administered as initial therapy with metformin improved glycaemic control and was well tolerated in patients with type 2 diabetes (10).

Sulphonylureas are among the most frequently prescribed and least costly oral antidiabetic drugs (OADs); their mechanism of action involves binding to the beta-cell sulphonylurea receptor 1, which ultimately stimulates insulin release (11,12). While specific guidelines vary, sulphonylureas are most often recommended as second-line therapy (after metformin) (13) and in some cases, as first-line OAD therapy in patients who are not overweight (12). Studies have demonstrated clinically significant improvements in glycaemic control with sulphonylurea therapy. However, as monotherapy, 5-year failure rates based on a fasting plasma glucose (FPG) > 10.0 mmol/l after at least 6 weeks of treatment at the maximum dose are approximately 34% (3,11). Sulphonylurea therapy is associated with limitations, including the potential for beta-cell toxicity, weight gain and an increased risk of hypoglycaemia with sulphonylurea-induced hyperinsulinaemia (5,11,14–16). Combination therapy of a DPP-4 inhibitor with a submaximal dose of a sulphonylurea represents an alternative treatment approach that may provide improved glycaemic control earlier in the disease course and allow the use of lower doses of sulphonylurea to reduce the risk for dose-related adverse events (AEs) (17,18).

The current trial evaluated the safety and efficacy of saxagliptin added to submaximal-dose sulphonylurea (glyburide) therapy, compared with uptitration of sulphonylurea (glyburide) monotherapy, in patients with type 2 diabetes and inadequate glycaemic control with submaximal-dose sulphonylurea therapy alone.

## Patients and methods

### Patients

Patients aged 18−77 years (inclusive) with type 2 diabetes and inadequate glycaemic control (HbA_1c_ screening value ≥ 7.5 to ≤ 10.0%) on a submaximal sulphonylurea dose [defined as less than the maximum approved dose for each sulphonylurea (see Table S1 for list of sulphonylureas and doses)] for ≥ 2 months before screening and with fasting C-peptide ≥ 1.0 ng/ml (0.3 nmol/l) and body mass index (BMI) ≤ 40 kg/m^2^ were eligible. Exclusion criteria included symptoms of poorly controlled diabetes; history of diabetic ketoacidosis or hyperosmolar non-ketotic coma; insulin therapy within 1 year of screening; cardiovascular event within 6 months of study entry or New York Heart Association stage III/IV congestive heart failure and/or known left ventricular ejection fraction ≤ 40%; significant history of renal or liver disease; psychiatric disorder; history of alcohol or drug abuse within the previous year; treatment with potent CYP 3A4 inhibitors or inducers; immunocompromised individuals; active liver disease or clinically significant abnormal hepatic, renal, endocrine, metabolic or haematological screening tests.

This study was conducted in accordance with Good Clinical Practice, as defined by the International Conference on Harmonisation, and in accordance with the ethical principles underlying the European Union Directive 2001/20/EC, the United States Code of Federal Regulations, Title 21, Part 50 (21CFR50), and the Declaration of Helsinki. Study protocol, amendments and patient informed consent were approved by the Institutional Review Board/Independent Ethics Committee at each site. All patients provided written, informed consent.

### Study design

This study (CV181-040) was a 24-week, phase 3, randomised, multicentre, 3-arm, double-blind, international trial. Patients were recruited from outpatient settings, advertisements, postings and referrals. Eligible patients entered a 4-week, single-blind, dietary and exercise placebo lead-in period during which they discontinued their current sulphonylurea therapy and received open-label glyburide 7.5 mg/day. Patients were instructed by a registered dietitian, registered nurse, physician, certified diabetes educator or nutritionist on diet and exercise in accordance with the American Diabetes Association (ADA) or similar local guidelines to be followed for the study duration. Good compliance (≥ 80 to ≤ 120%) with placebo was required to be eligible for randomisation before the short-term treatment period. Patients with an HbA_1c_ level ≥ 7.0% and mean FPG (MFPG) or FPG ≥ 140 mg/dl (7.8 mmol/l) or mean fasting whole blood glucose (MFWBG) ≥ 131 mg/dl (7.3 mmol/l) continued treatment with open-label glyburide 7.5 mg/day and were randomised (1 : 1 : 1) via Interactive Voice Response System to one of three treatment groups (block size 3) utilising a double-dummy design: saxagliptin 2.5 mg/day (saxagliptin 2.5 mg + glyburide), saxagliptin 5 mg/day (saxagliptin 5 mg + glyburide) or placebo + blinded glyburide 2.5 mg/day [uptitrated glyburide; initial total daily dose (TDD) of glyburide 10 mg]. A one-time decrease in open-label glyburide to 5 mg/day was permitted at the investigator’s discretion for patients who developed hypoglycaemia. Uptitration of blinded glyburide was permitted at weeks 2 and 4 in the uptitrated glyburide treatment group for patients satisfying prespecified glycaemic criteria [MFPG ≥ 100 mg/dl (5.5 mmol/l) or MFWBG ≥ 95 mg/dl (5.3 mmol/l)] to a maximum TDD of 15 mg (7.5 mg open-label + 7.5 mg blinded glyburide), provided the open-label glyburide dose had not been previously decreased because of hypoglycaemia. Throughout the study, double-blind study medication was to be taken twice daily, before the morning and evening meals to allow the glyburide dose to be split between morning and evening. Saxagliptin was to be taken in the morning. Patients were eligible for rescue therapy based on progressively strict glycaemic control criteria over 24 weeks if MFPG levels were: > 240 mg/dl (13.3 mmol/l) (weeks 4 and 6); > 220 mg/dl (12.2 mmol/l) (week 8); and > 200 mg/dl (11.1 mmol/l) (weeks 12, 16, 20 and 24), or if MFWBG > 221 mg/dl (12.3 mmol/l) (weeks 4 and 6); > 203 mg/dl (11.3 mmol/l) (week 8); or > 185 mg/dl (10.3 mmol/l) (weeks 12, 16, 20 and 24). Patients meeting rescue criteria entered into the long-term extension period, during which they were administered open-label metformin and glyburide in addition to blinded study medication. Patients completing 24 weeks of treatment without rescue also entered the long-term extension period. Long-term extension results will be reported in a future communication.

### Study end-points

#### Efficacy assessments

The primary efficacy end-point was HbA_1c_ change from baseline to week 24. Secondary efficacy end-points assessed at week 24 and listed in the order tested were change from baseline in FPG, proportion of patients achieving HbA_1c_ < 7.0%, and change from baseline in postprandial glucose (PPG) area under the curve (AUC) from 0 to 180 min in response to a 75-g oral glucose tolerance test (OGTT) with samples drawn at time −30 min, immediately prior to time 0 min, and at +30, +60, +120 and +180 min after oral glucose ingestion. Other efficacy end-points included the proportion of patients achieving HbA_1c_ ≤ 6.5%; change from baseline to week 24 in fasting insulin, C-peptide and glucagon; postprandial insulin, C-peptide and glucagon AUC; beta-cell function [measured by homeostatic model assessment (HOMA)-2β]; insulin resistance (measured using HOMA-2IR); the proportion of patients requiring rescue for failing to achieve prespecified glycaemic targets or discontinuing for lack of efficacy; the proportion of patients achieving a glycaemic response at week 24 based on prespecified criteria; and PPG-AUC at the 120-min time point. Changes from baseline to week 24 in body weight, lipid parameters, insulinogenic index, Matsuda index (19) and oral glucose insulin sensitivity (OGIS) (20) were also examined. Subgroup analyses for baseline HbA_1c_ were prespecified.

#### Safety assessments

Safety and tolerability assessments included incidence of AEs, serious AEs (SAEs), AE-related discontinuations as well as results for electrocardiograms, vital signs and clinical laboratory tests. AEs of hypoglycaemia and confirmed hypoglycaemia, defined as symptoms of hypoglycaemia with a fingerstick glucose ≤ 2.8 mmol/l, were also recorded.

### Statistical analyses

Efficacy analyses were conducted utilising data collected at baseline and postbaseline in the Randomised Patients data set, which consisted of all randomised patients who took at least one dose of double-blind study medication. An ANCOVA was performed on continuous efficacy end-points using last-observation-carried-forward (LOCF) methodology with treatment group as an effect and baseline value as the covariate. Within the framework of the ANCOVA model, point estimates and 95% confidence intervals were calculated for mean changes between each of the saxagliptin treatment groups and the uptitrated glyburide group. For the primary end-point, each comparison between a saxagliptin treatment group and the uptitrated glyburide group was performed at the 0.027 alpha level from Dunnett’s adjustment so that the overall (family-wise) type I error rate was controlled at the 0.05 significance level. Sequential testing methodology was used for secondary efficacy end-points. At each step in the testing sequence, only the saxagliptin treatment groups that were significantly superior to uptitrated glyburide were tested at the subsequent step. Summaries of categorical end-points such as the percentage of patients achieving a therapeutic glycaemic response at week 24, the proportion of patients requiring rescue/discontinuation because of lack of glycaemic control and the proportion of patients with reported and confirmed hypoglycaemia included frequencies and percentages for each treatment group; treatment groups were compared using the two-sided Fisher exact test. Demographic and other baseline characteristics were summarised using descriptive statistics by treatment group. LOCF methodology was used to handle missing data. The adjusted mean changes from baseline within each treatment group as well as the difference in mean change from baseline between each treatment group and the placebo or active comparator treatment group for each subgroup were calculated, as well as the corresponding subgroup by treatment interaction p-value. Systeme International (SI) conversion from mg/dl to mmol/l of glucose was calculated with the equation: mg/dl × 0.0555. All other SI conversions pertaining to data presented for this study are noted in Table S2. Estimated average glucose (eAG) values were calculated *post hoc* based on HbA_1c_ values using the linear regression: eAG_mg/dl_ = 28.7 × HbA_1c_− 46.7 (21).

Safety analyses were performed in the treated patient population, consisting of patients who received at least one dose of study medication. Events of hypoglycaemia and confirmed hypoglycaemia were recorded and analysed separately from other AEs. Hypoglycaemic event intensity was graded according to the investigator’s discretion, as were all other AEs. Efficacy and safety measurements obtained after rescue were not included in analyses.

Based on the primary end-point, the sample size afforded at least 90% power to detect a difference in HbA_1c_ means of 0.4% between each saxagliptin treatment group and the uptitrated glyburide treatment group, assuming a standard deviation (SD) of 1.2%.

## Results

### Disposition, baseline demographics and disease characteristics

**Figure 1:**
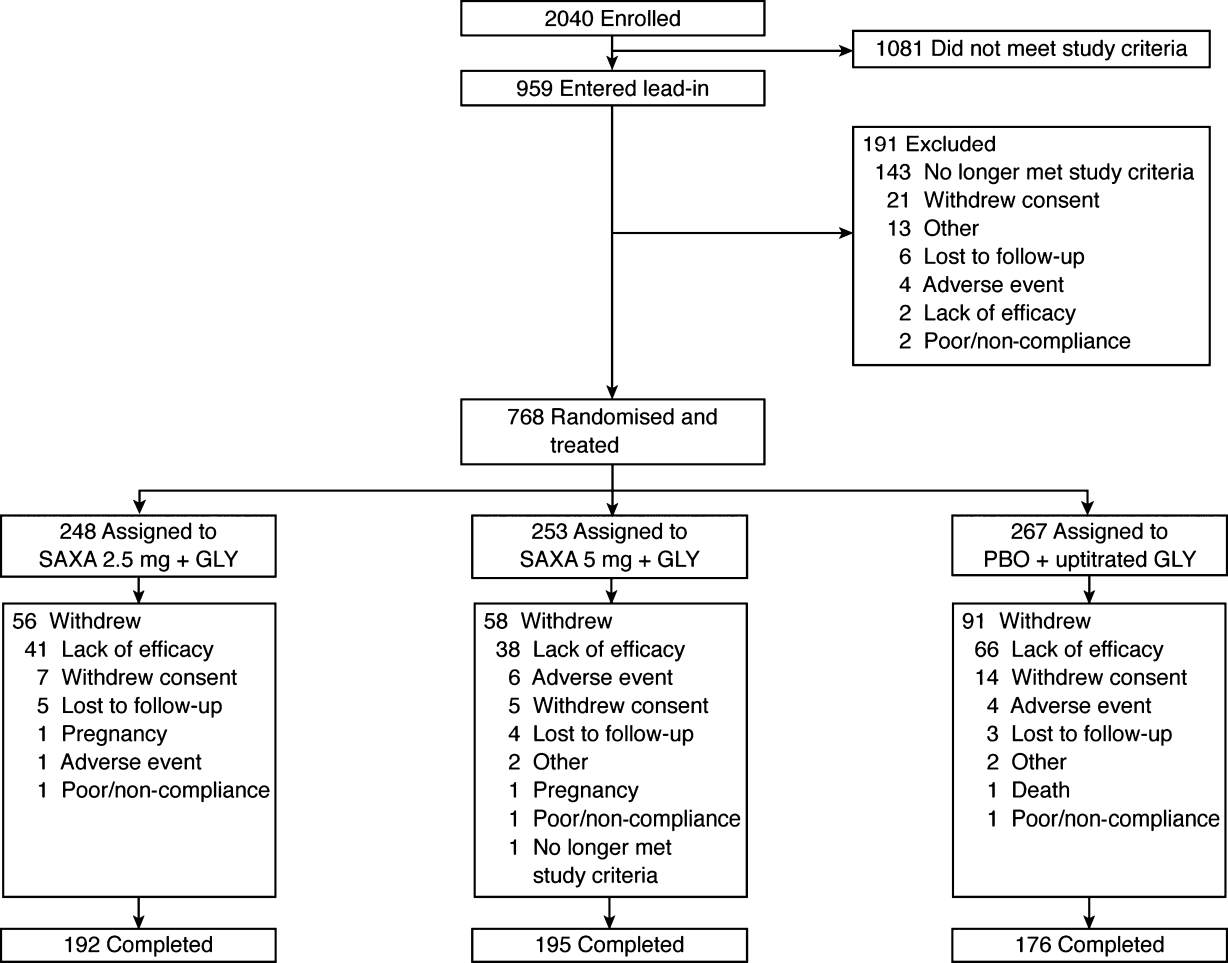
Flow of patients through the study. Recruitment period ran from 17 April 2006 through 2 February 2007, with follow up ending on 14 September 2007. SAXA 2.5 mg + GLY = saxagliptin 2.5 mg/day plus open-label glyburide 7.5 mg/day. SAXA 5 mg + GLY = saxagliptin 5 mg/day plus open-label glyburide 7.5 mg/day. PBO + uptitrated GLY = placebo plus double-blind glyburide 2.5 mg/day and open-label glyburide 7.5 mg/day

A total of 768 patients were randomised and treated with double-blind therapy; 563 patients completed the 24-week treatment period (Figure 1). Demographic and baseline clinical characteristics were generally well balanced across all treatment groups (Table 1). The mean (SD) duration of the previous sulphonylurea treatment was 2.5 (3.54), 2.3 (2.96) and 2.4 (3.60) years in the saxagliptin 2.5- and 5-mg, and uptitrated glyburide groups, respectively. All patients had been previously treated with a sulphonylurea before study entry; 64% of patients received prior glyburide treatment.

**Table 1:** Baseline demographic and clinical characteristics by randomised group

	SAXA 2.5 mg + GLY	SAXA 5 mg + GLY	PBO + UPGLY
Characteristic	(*n =*248)	(*n =*253)	(*n =*267)
Age (years)*	55.4 (9.6)	54.9 (10.0)	55.1 (10.7)
Age ≥ 65 (years)†	43 (17.3)	42 (16.6)	52 (19.5)
Gender†			
Men	113 (45.6)	110 (43.5)	123 (46.1)
Women	135 (54.4)	143 (56.5)	144 (53.9)
Race†,‡			
White	148 (59.7)	151 (59.7)	152 (56.9)
Black/African American	5 (2.0)	7 (2.8)	7 (2.6)
Asian	42 (16.9)	46 (18.2)	51 (19.1)
Other	53 (21.4)	49 (19.4)	57 (21.3)
Weight (kg)*	75.2 (14.4)	76.2 (17.6)	75.6 (17.4)
BMI (kg/m^2^)*	29.1 (4.5)	29.2 (4.6)	28.8 (4.7)
Duration of diabetes (years)*	7.1 (5.9)	6.8 (5.8)	6.8 (5.7)
HbA_1c_ (%)*	8.4 (0.9)	8.5 (0.9)	8.4 (0.9)
< 8%†	88 (35.5)	74 (29.2)	93 (34.8)
≥ 8 to < 9%†	101 (40.7)	102 (40.3)	99 (37.1)
≥ 9%†	59 (23.8)	76 (30.0)	75 (28.1)
Not reported	0 (0)	1 (0.4)	0 (0)
FPG (mg/dl)*	170 (41.9)	175 (44.3)	175 (42.8)

BMI, body mass index; FPG, fasting plasma glucose.

* Values are expressed as mean (SD).

† Values are expressed as *n* (%).

‡ Race/ethnicity was self-reported. SAXA 2.5 mg + GLY = saxagliptin 2.5 mg/day plus open-label glyburide 7.5 mg/day. SAXA 5 mg + GLY = saxagliptin 5 mg/day plus open-label glyburide 7.5 mg/day. PBO + UPGLY = placebo plus double-blind glyburide 2.5 mg/day and open-label glyburide 7.5 mg/day.

Saxagliptin-treated patients received a lower final mean TDD (open-label and blinded) of glyburide vs. the uptitrated glyburide group. Approximately, 92% of patients in the uptitrated glyburide group were titrated to the maximum daily glyburide dose (15 mg). The final mean (SD) glyburide TDD was 7.4 (0.5) mg (saxagliptin 2.5 mg), 7.4 (0.6) mg (saxagliptin 5 mg) vs. 14.6 (1.3) mg (uptitrated glyburide group). Open-label glyburide was downtitrated to 5 mg/day in 4.0%, 5.1% and 2.2% of patients in the saxagliptin 2.5- and 5-mg and uptitrated glyburide groups, respectively, primarily in response to hypoglycaemia-related AEs.

### Efficacy

**Figure 2:**
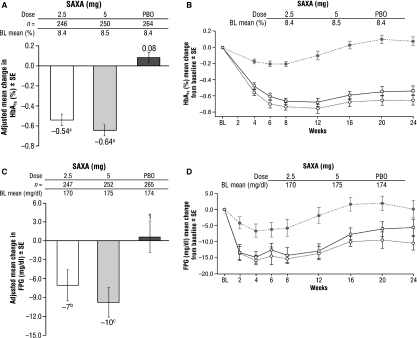
Changes in glycaemic variables during 24-week treatment period: saxagliptin + MET vs. monotherapy. (A) HbA_1c_ adjusted mean change from baseline to week 24. (B) HbA_1c_ mean change from baseline during 24-week treatment period. (C) Fasting plasma glucose (FPG) adjusted mean change from baseline to week 24. (D) FPG mean change from baseline during 24-week treatment period. Open bars (A and C) and open squares (B and D)*,* saxagliptin 2.5 mg + GLY; grey bars (A and C), and open circles (B and D)*,* saxagliptin 5 mg + GLY; dark grey bars (A and C) and shaded circles (B and D), placebo + UPGLY. ^a^p < 0.0001; ^b^p = 0.0218; ^c^p = 0.0020

At 24 weeks, patients randomised to saxagliptin 2.5 or 5 mg demonstrated statistically significant reductions in HbA_1c_, FPG and PPG-AUC from baseline, compared with patients in the uptitrated glyburide group. Baseline vs. week 24 HbA_1c_ mean values were 8.4% vs. 7.8%; 8.5% vs. 7.8% and 8.4% vs. 8.5% for saxagliptin 2.5 and 5 mg and uptitrated glyburide respectively. Corresponding eAG values were 194 vs. 177 mg/dl (10.8 vs. 9.8 mmol/l) for saxagliptin 2.5 mg; 197 vs. 177 mg/dl (10.9 vs. 9.8 mmol/l) for saxagliptin 5 mg; and 194 vs. 197 mg/dl (10.8 vs. 10.9 mmol/l) for uptitrated glyburide. Adjusted mean change in HbA_1c_ from baseline was −0.54% and −0.64% for saxagliptin 2.5 and 5 mg vs. +0.08% for uptitrated glyburide (both p < 0.0001) (Figure 2A). Greater HbA_1c_ mean reductions were observed with saxagliptin therapy vs. uptitrated glyburide at week 4, the earliest time point assessed for HbA_1c_, and persisted at all subsequent time points (Figure 2B). The greatest HbA_1c_ reductions were demonstrated in the saxagliptin 5-mg treatment group.

Statistically significantly greater mean reductions in FPG at week 24 were observed for saxagliptin 2.5 (p = 0.0218) and 5 mg (p = 0.002) vs. uptitrated glyburide. Adjusted mean change from baseline was −7 mg/dl (−0.40 mmol/l) (saxagliptin 2.5 mg) and −10 mg/dl (−0.50 mmol/l) (saxagliptin 5 mg) vs. +1 mg/dl (+0.04 mmol/l) for uptitrated glyburide (Figure 2C). Reductions in MFPG values were apparent by week 2, the earliest time point for FPG assessment (Figure 2D).

The proportion of patients achieving an HbA_1c_< 7.0% at week 24 was statistically significantly greater for saxagliptin 2.5 mg (22.4%) and saxagliptin 5 mg (22.8%) vs. uptitrated glyburide (9.1%; both p < 0.0001). The proportion of patients achieving an HbA_1c_ ≤ 6.5% at week 24 was statistically significantly greater for saxagliptin 5 mg (10.4%) vs. uptitrated glyburide (4.5%; p = 0.0117).

**Figure 3:**
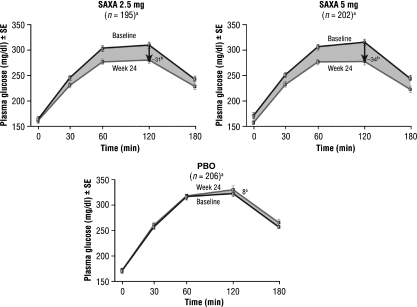
Postprandial glucose response to 3-h OGTT: baseline vs. week 24. Black line with squares, baseline values at 0, +30, +60, +120 and +180-min time points; grey line with squares, week 24 values at 0, +30, +60, +120 and +180-min time points. ^a^Sample size at 120-min time point; ^b^adjusted mean change in 120-min PPG

A statistically significant reduction in glucose exposure from baseline to week 24 was seen in PPG-AUC during the OGTT for the saxagliptin treatment groups vs. uptitrated glyburide [−4296 mg·min/dl (−238 mmol·min/l) and −5000 mg·min/dl (−278 mmol·min/l) vs. +1196 mg·min/dl (+66 mmol·min/l) respectively, both p < 0.0001 vs. uptitrated glyburide]. An overall decrease from baseline in mean glucose concentration at all time points of the OGTT occurred in both saxagliptin treatment groups compared with increases in the uptitrated glyburide group at week 24 (Figure 3). At the 120-min time point of the OGTT, PPG adjusted mean changes from baseline were −31 mg/dl (−2 mmol/l) for saxagliptin 2.5 mg and −34 mg/dl (−2 mmol/l) for saxagliptin 5 mg relative to +8 mg/dl (+0.4 mmol/l) for uptitrated glyburide, both p < 0.0001.

Changes in other efficacy assessments are listed in Table 2. At week 24, saxagliptin treatment increased postprandial insulin and C-peptide AUC to a greater degree than did uptitrated glyburide. Saxagliptin treatment did not have an effect on fasting insulin or C-peptide. Postprandial glucagon AUC was decreased to a greater degree with saxagliptin treatment vs. uptitrated glyburide; decreases in fasting glucagon were only noted in the saxagliptin 5-mg group [−0.8 pg/ml (−0.8 ng/l)] vs. uptitrated glyburide [+4.5 pg/ml (+4.5 ng/l)]. There was no change in beta-cell function (HOMA-2β assessment) at week 24 in the saxagliptin treatment groups vs. uptitrated glyburide. Similarly, saxagliptin treatment was not associated with changes in early insulin response (as measured using the 30-min insulinogenic index), insulin sensitivity (based on OGIS and Matsuda indices) or insulin resistance (HOMA-2IR assessment) vs. uptitrated glyburide. Mean body weight increased in all treatment groups; adjusted mean increases were statistically significantly greater in each saxagliptin treatment group vs. uptitrated glyburide [+0.7 kg (p = 0.0381) and +0.8 kg (p = 0.0120) for saxagliptin 2.5 and 5 mg respectively, vs. +0.3 kg for uptitrated glyburide]. Saxagliptin had no clear effect on mean fasting total cholesterol levels. All three treatment groups experienced small numerical increases in triglycerides and low-density lipoprotein-cholesterol and numerical decreases in high-density lipoprotein-cholesterol. HbA_1c_ reductions were similar, regardless of duration of diabetes, geographic region, race, gender, age, ethnicity or BMI. No interaction of treatment with baseline HbA_1c_ was observed (p = 0.5907).

**Table 2:** Other efficacy assessments at 24 weeks

	*n*	Baseline mean ± SE	Week 24 mean ± SE	Adjusted mean change from baseline ± SE	95% CI
**PP insulin AUC (μU·min/ml)**					
SAXA 2.5 + GLY	184	6220 ± 293.7	7381 ± 340.8	1174 ± 211.8	(758, 1590)
SAXA 5 + GLY	192	5889 ± 227.0	7042 ± 292.6	1071 ± 207.5	(663, 1478)
PBO + UPGLY	200	6400 ± 373.6	5710 ± 269.6	−624 ± 203.2	(−1023, −224)
**PP glucagon AUC (pg·min/ml)**					
SAXA 2.5 + GLY	183	13153 ± 388.2	12936 ± 377.4	−125 ± 275.5	(−667, 416)
SAXA 5 + GLY	188	12443 ± 321.3	12101 ± 312.3	−566 ± 272.2	(−1101, −32)
PBO + UPGLY	193	13244 ± 377.2	13884 ± 324.8	772 ± 268.3	(244, 1299)
**PP C-peptide AUC (ng·min/ml)**					
SAXA 2.5 + GLY	163	1103 ± 32.7	1205 ± 32.8	107 ± 18.3	(71, 143)
SAXA 5 + GLY	161	1097 ± 33.9	1206 ± 35.1	113 ± 18.5	(77, 149)
PBO + UPGLY	175	1042 ± 30.8	1044 ± 27.1	−6 ± 17.7	(−41, 29)
**HOMA-2β (%)**					
SAXA 2.5 + GLY	236	65.3 ± 2.18	74.4 ± 2.59	9.5 ± 2.32	(4.9, 14.0)
SAXA 5 + GLY	246	64.1 ± 2.23	71.8 ± 2.86	7.6 ± 2.27	(3.2, 12.1)
PBO + UPGLY	257	62.9 ± 2.29	67.9 ± 2.75	4.6 ± 2.22	(0.2, 8.9)
**HOMA-2IR (no unit)**					
SAXA 2.5 + GLY	236	3.04 ± 0.095	3.14 ± 0.079	0.09 ± 0.067	(−0.04, 0.22)
SAXA 5 + GLY	246	3.14 ± 0.095	3.00 ± 0.086	−0.10 ± 0.065	(−0.23, 0.02)
PBO + UPGLY	257	3.01 ± 0.083	3.19 ± 0.086	0.15 ± 0.064	(0.03, 0.28)
**OGIS (ml/min·m^2^)**				**Unadjusted mean change from baseline ± SE**	
SAXA 2.5 + GLY	182	293.4 ± 3.93	284.5 ± 4.22	−8.9 ± 4.74	(−18.2, 0.5)
SAXA 5 + GLY	187	293.9 ± 4.28	290.0 ± 4.27	−3.9 ± 4.69	(−13.1, 5.4)
PBO + UPGLY	195	285.1 ± 4.14	285.9 ± 4.89	0.7 ± 4.67	(−8.5, 10.0)
**Matsuda index (no unit)**					
SAXA 2.5 + GLY	176	3.67 ± 0.225	3.24 ± 0.207	−0.44 ± 0.181	(−0.79, −0.08)
SAXA 5 + GLY	179	3.44 ± 0.179	3.22 ± 0.154	−0.22 ± 0.142	(−0.51, 0.06)
PBO + UPGLY	188	3.51 ± 0.219	3.30 ± 0.169	−0.21 ± 0.156	(−0.52, 0.10)
**Insulinogenic index (no unit)**					
SAXA 2.5 + GLY	180	0.29 ± 0.131	0.26 ± 0.030	−0.03 ± 0.132	(−0.29, 0.23)
SAXA 5 + GLY	186	0.22 ± 0.069	0.34 ± 0.103	0.12 ± 0.122	(−0.12, 0.36)
PBO + UPGLY	194	0.16 ± 0.013	0.16 ± 0.020	−0.00 ± 0.022	(−0.05, 0.04)

AUC, area under the curve; CI, confidence interval; HDL, high-density lipoprotein; HOMA, homeostatic model assessment; LDL, low-density lipoprotein; OGIS, oral glucose insulin sensitivity; PP, postprandial; SE, standard error. SAXA 2.5 mg + GLY = saxagliptin 2.5 mg/day plus open-label glyburide 7.5 mg/day. SAXA 5 mg + GLY = saxagliptin 5 mg/day plus open-label glyburide 7.5 mg/day. PBO + UPGLY = placebo plus double-blind glyburide 2.5 mg/day and open-label glyburide 7.5 mg/day.

The proportion of patients (*n*/*N*) discontinuing for lack of glycaemic control or rescued for meeting prespecified glycaemic criteria was lower for saxagliptin 2.5 mg [18.1% (45/248)] and 5 mg [16.6% (42/253)] vs. uptitrated glyburide [29.6% (79/267)].

### Safety and tolerability

Overall, saxagliptin added to submaximal glyburide therapy was generally well tolerated. The proportion of patients reporting any AE was similar across all treatment groups, with no evidence of a dose–response relationship (Table 3). One death (sudden cardiac death) occurred in the uptitrated glyburide group. The majority of AEs were mild or moderate in intensity.

**Table 3:** Safety and tolerability during 24-week treatment period by randomised group

	SAXA 2.5 mg + GLY (*n =*248)	SAXA 5 mg + GLY (*n =*253)	PBO + UPGLY (*n =*267)
Adverse events (%)*			
≥ 1 AE	186 (75.0)	183 (72.3)	205 (76.8)
≥ 1 related AE	49 (19.8)	54 (21.3)	38 (14.2)
Discontinuation due to AE	3 (1.2)	8 (3.2)	4 (1.5)
≥ 1 SAE†	4 (1.6)	6 (2.4)	6 (2.2)
≥ 1 related SAE	0	0	0
Discontinuation due to SAEs	0	1 (0.4)	1 (0.4)
Deaths	0	0	1 (0.4)
**Adverse events (≥ 5%)‡,§**			
Urinary tract infection	13 (5.2)	27 (10.7)	22 (8.2)
Nasopharyngitis	14 (5.6)	15 (5.9)	18 (6.7)
Upper respiratory tract infection	11 (4.4)	16 (6.3)	18 (6.7)
Influenza	13 (5.2)	10 (4.0)	16 (6.0)
Diarrhoea	14 (5.6)	10 (4.0)	14 (5.2)
Back pain	12 (4.8)	15 (5.9)	12 (4.5)
Pain in extremity	11 (4.4)	9 (3.6)	15 (5.6)
Headache	19 (7.7)	19 (7.5)	15 (5.6)
Cough	13 (5.2)	10 (4.0)	13 (4.9)
Hypertension	9 (3.6)	16 (6.3)	6 (2.2)
Reported hypoglycaemia¶	33 (13.3)**	37 (14.6)††	27 (10.1)
Confirmed hypoglycaemia‡‡	6 (2.4)§§	2 (0.8)¶¶	2 (0.7)

* AE defined as any new or worsening illness, sign, symptom, or clinically significant laboratory test abnormality as noted by the investigator during the course of the study, regardless of the investigator’s attribution of the event to study treatment.

† SAE defined as an AE that was fatal, life threatening, required in-patient hospitalisation or prolonged an existing hospitalisation, resulted in persistent or significant disability or incapacity, a cancer, a congenital anomaly/birth defect, resulted in the development of drug dependency or drug abuse, or was an important medical event that jeopardised the patient or required intervention to prevent a serious outcome.

‡ Excludes hypoglycaemia.

§ AEs outside of hypoglycaemia were not tested for statistical significance vs. comparator.

¶ Reported hypoglycaemia was defined as events consistent with signs or symptoms of hypoglycaemia with or without documented blood glucose levels.

** p = 0.2741 vs. PBO + UPGLY.

†† p = 0.1417 vs. PBO + UPGLY.

‡‡ Confirmed hypoglycaemia was defined by a fingerstick glucose value ≤ 50 mg/dl (2.8 mmol/l) with associated symptoms.

§§ p = 0.1626 vs. PBO + UPGLY.

¶¶ p = 1.0000 vs. PBO + UPGLY.

The proportion of patients with skin-related AEs was: 8.9% [22/248 (saxagliptin 2.5 mg)], 4.7% [12/253 (saxagliptin 5 mg)] and 4.9% [13/267 (uptitrated glyburide)], with no evidence of a dose-related effect. AEs related to localised oedema were reported in two saxagliptin-treated patients: one in the saxagliptin 2.5-mg group and one in the saxagliptin 5-mg group; none were reported in the uptitrated glyburide treatment group. Both events were reported to be of mild intensity and not related to study drug, and neither led to discontinuation. No events of Stevens-Johnson syndrome or angio-oedema were reported. Cardiac disorder events were: 2.0% (5/248) for saxagliptin 2.5 mg, 4.0% (10/253) for saxagliptin 5 mg and 3.7% (10/267) for uptitrated glyburide. AEs of hypertension were reported for 9/248 (3.6%, saxagliptin 2.5 mg), 16/253 (6.3%, saxagliptin 5 mg) and 6/267 (2.2%, uptitrated glyburide) patients; however, mean systolic (SBP) and diastolic blood pressure (DBP) decreased in all treatment groups. The mean change from baseline at week 24 for saxagliptin 2.5 and 5 mg, and uptitrated glyburide was −3.9, −3.2 and −2.0 mmHg (SBP), and −3.3, −1.8 and −2.4 mmHg (DBP) respectively. There were no clinically meaningful drug effects on any other laboratory safety parameter.

There was no statistically significant difference in the incidence of reported and confirmed hypoglycaemic events in the saxagliptin 2.5- and 5-mg treatment groups vs. the uptitrated glyburide treatment group (Table 3). Confirmed hypoglycaemia occurred in 6/248 (2.4%), 2/253 (0.8%) and 2/267 (0.7%) of patients in the saxagliptin 2.5- and 5-mg treatment groups vs. the uptitrated glyburide treatment group. Of the 10 confirmed hypoglycaemic events, most were of mild or moderate intensity; a single hypoglycaemia-related AE judged to be severe in intensity by the study investigator occurred in a patient receiving saxagliptin 2.5 mg, which was easily managed by the patient. No hypoglycaemic event was judged by the study investigator to be an SAE or led to discontinuation of study therapy.

## Discussion

The therapeutic goal for the treatment of type 2 diabetes is to achieve and maintain glycaemic levels without compromising safety or tolerability (22,23). This study demonstrated that in patients with type 2 diabetes not achieving glycaemic control on glyburide monotherapy, the addition of saxagliptin once daily to submaximal doses of glyburide for 24 weeks provided statistically significant and clinically meaningful reductions in key parameters of glycaemic control vs. uptitrated glyburide, without statistically significantly increasing the frequency of hypoglycaemia. Reductions observed in HbA_1c_ with saxagliptin added to submaximal glyburide corresponded to substantial improvements in FPG, the proportion of patients reaching goal, and PPG-AUC, with maximal benefits observed in the saxagliptin 5-mg group. These 24-week results are in contrast to increases in HbA_1c_, FPG and PPG-AUC in the uptitrated glyburide group, indicating that the addition of saxagliptin to submaximal glyburide therapy is preferable to continued monotherapy with higher doses of sulphonylurea.

HbA_1c_ reductions achieved with saxagliptin added to submaximal glyburide therapy occurred early. Decreases relative to uptitrated glyburide were noted by week 4 and were maintained throughout the remainder of the treatment period. Importantly, the effect of saxagliptin on HbA_1c_ lowering was consistent across prespecified subgroups including age, gender, ethnicity, BMI, geographic distribution and duration of diabetes, suggesting its appropriateness for use in a variety of patients with type 2 diabetes.

More than twice as many patients treated with saxagliptin 2.5 and 5 mg achieved HbA_1c_ goals recommended by the ADA and European Association for the Study of Diabetes (< 7.0%) vs. patients treated with uptitrated glyburide (24). Similarly, more than twice as many patients treated with saxagliptin 5 mg achieved the American Association of Clinical Endocrinologists’ recommended goal of HbA_1c_≤ 6.5%, compared with patients treated with uptitrated glyburide (25). Patients treated with saxagliptin achieved HbA_1c_ targets of < 7% more rapidly than patients receiving uptitrated glyburide, potentially reducing the harmful impact of glucotoxicity on beta-cell function earlier in the disease course (26).

Relative to uptitrated glyburide, saxagliptin treatment led to an increase in postprandial insulin. A possible explanation for this observation is that the maximal insulin-releasing effect of a sulphonylurea may be enhanced by the addition of another OAD with a complementary mechanism of action also known to increase insulin levels or beta-cell function. DPP-4 inhibitors enhance GLP-1 and GIP levels, thereby acting to promote insulin synthesis and release and improve beta-cell function, suggesting their utility in combination with sulphonylurea therapy.

Saxagliptin 2.5 and 5 mg were generally well tolerated, with no clinically meaningful difference between treatment groups in the proportion of patients reporting AEs. There was no evidence of a dose-response relationship for AEs. Combination therapy is often associated with an increased risk for hypoglycaemia, particularly combinations that use sulphonylureas or insulin (27). Treatment with saxagliptin did not statistically significantly increase the frequency of hypoglycaemia, compared with glyburide uptitration, even with significantly greater glycaemic efficacy achieved with add-on therapy. Despite no observed statistically significant increase in hypoglycaemia in this study, these results must be viewed with appropriate caution given that hypoglycaemia is a concern in any sulphonylurea-based therapeutic regimen.

An increase from baseline in body weight, of small magnitude and unclear clinical relevance, occurred in all treatment groups; however, weight gain was higher in the saxagliptin treatment groups. Improved glycaemic control has been shown to promote weight gain in certain instances by decreasing glucosuria (13,28). A possible explanation is that the weight gain in the saxagliptin treatment groups was a result of decreased glucosuria, whereas sulphonylurea-induced weight gain may have been mitigated by the glucosuria caused by inadequate glycaemic control in the uptitration arm.

Only data collected prior to rescue were used for efficacy and safety analyses, which could be a study limitation in that experience following rescue therapy is not included in the analyses. However, this approach was taken to minimise the potential confounding of rescue therapy on both safety and efficacy parameters.

In patients with type 2 diabetes and inadequate glycaemic control, initiating therapy with a sulphonylurea and uptitrating the dosage until maximum concentrations are reached is an approach commonly used in clinical practice in certain regions. The addition of saxagliptin to submaximal sulphonylurea treatment – as opposed to uptitration – offers a potentially improved strategy for achieving tighter glycaemic control and contrasts with existing ‘treat-to-failure’ approaches, in which clinical inertia often leads to an unacceptable glycaemic burden (29). Other randomised controlled studies of oral antidiabetic agents in combination with submaximal sulphonylurea therapy have also demonstrated improved glycaemic control, compared with uptitration of sulphonylurea monotherapy (30,31). Further, the addition of saxagliptin to submaximal sulphonylurea therapy has the potential to yield additional benefits, such as preservation of beta-cell function and reduction of potentially toxic beta-cell effects associated with maximal sulphonylurea therapy (15,16).

## Conclusions

The glycaemic benefits demonstrated in this study support the use of the DPP-4 inhibitor saxagliptin as add-on therapy to glyburide or other sulphonylurea agents in patients with type 2 diabetes and inadequate glycaemic control with sulphonylurea monotherapy. The favourable tolerability profile of combination therapy assessed in the current trial indicates that the addition of saxagliptin may be appropriate when a submaximal dose of glyburide or other sulphonylurea provides inadequate glycaemic control.

## Appendix

CV181-040 study investigators:

***Argentina:*** Fideleff H, Maffei L, Migueles J, Sposetti G, Ulla MR, Waitman J; ***Brazil:*** Chacra AR, Eliaschewitz F, Felicio J, Forti A, Gross JL, Hissa MN, Purish S, Repetto G, Saraiva JF, Sgarbi JA; ***Hong Kong:*** Lee KF, Tong P; ***Israel:*** Adawi F, Buchs A, Cohen J, Harman Bohem I, Karnieli E, Klainman E, Raz I, Wainstein J, Yerushalmy Y; ***Mexico:*** Aguilera M, Alvarado R, Calvo C, Gonzalez G, Gonzalez Gonzalez G, Jerjes-Sanchez C, Medina Pech CE, Robles FJ, Saldate C, Sauque Reyna L; ***Peru:*** Caballero J, Villena J, Zapata L, Zubiate C; ***Philippines:*** Jasul G, Lim-Abrahan MA, Pacheco E, Tan GH; ***Puerto Rico:*** Abreu-Feshold F, Barranco Santana E, Claudio J, Vazquez Tanus JB; ***Republic of Korea:*** Ahn CW, Jang HC, Lee HC, Lee KW, Lee MK, Park JS, Woo JT, Yoo SJ, Yoon KH; ***Singapore:*** Lee KO; ***South Africa:*** Bernhardi D, Bhana SA, Burgess L, Chetty S, Distiller L, Kaplan H, Khutsoane DT, Moore R, Mynhardt JH; ***Taiwan:*** Chuang LM, Ho LT, Huang CN, Lin KC; ***Thailand:*** Shih KC; ***United States:*** Acampora MD, Adams J, Arnold G, Aventa A, Azorr M, Barrera JE, Bedel G, Bolick C, Brautigam D, Brinson AC, Catlett D, Chappel C, Chrysant S, Cohen K, Corbett III B, Damian D, Dass B, Diederich CF, Doolan R, Dyck D, Eberly J, Ellis C, Ganong KD, Goldberg R, Gray WL, Guthrie R, Harrison B, Hendley L, Hershberger VJ, Hoekstra JA, Hoyte S, Hurley S, Isakov T, Ison R, Jacks WP, Jain RK, Johnson D, Joiner J, Kayne D, Kayota SW, Landgarten S, Latham G, Lee FJ, Lenhard J, Lockwood RJ, Lucas KJ, McKeown-Biagas C, Mercado A, Michael B, Miller AB, Miller S, Mitchell JR, Montoro R, Morales L, Nagaria A, Norwood Jr P, Nurnberg RD, Patel M, Pearlstein P, Pearson D, Pudi KK, Rhudy J, Rigonan K, Ryan WM, Saway W, Schwartz SL, Shealy N, Sher L, Shue RG, Simon H, Sloan GK, Snyder B, Soufer J, Spence JA, Stevens JA, Sugimoto DH, Tarshis G, Trevino M, Wayne J, Weisbrot AJ, Wiegmann T, Witkin DB.

## Supporting Information

**Table S1** Eligible sulphonylureas and doses.

**Table S2** Conversion chart: conventional to SI units.
