# Supplementary material for: Saxagliptin added to a submaximal dose of sulphonylurea improves glycaemic control compared with uptitration of sulphonylurea in patients with type 2 diabetes: a randomised controlled trial
Source: Int J Clin Pract. 2009 Sep;63(9):1395–406. doi: 10.1111/j.1742-1241.2009.02143.x (PMC2779994; doi:10.1111/j.1742-1241.2009.02143.x)
Supplement: Supplementary file 1 [file ijcp0063-1395-SD1.doc]

**SUPPLEMENTARY MATERIAL**

**Table 1** Eligible Sulphonylureas and Doses

| **Sulphonylurea** | **Dose** |
| --- | --- |
| Acetohexamide | 500–1250 mg |
| Carbutamide | 500–750 mg |
| Chlorpropamide | 250–500 mg |
| Glibornurid | 25–50 mg |
| Gliciclamide | 500–1000 mg |
| Gliclazide | 80–240 mg  30–90 mg (modified release) |
| Glimepiride | 2–6 mg |
| Glipizide | 10–30 mg  5–15 mg (extended release) |
| Gliquidone | 45–135 mg |
| Glisentide | 5–15 mg |
| Glyburide/Glibenclamide | 5–15 mg (non-micronized)  3–9 mg (micronized) |
| Tolazamide | 250–750 mg |
| Tolbutamide | 1000–2500 mg |

**SUPPLEMENTARY MATERIAL**

**Table 2** Conversion Chart: Conventional to SI Units^a^

| **Component** | **Conventional Unit** | **Conversion Factor** | **SI Unit** |
| --- | --- | --- | --- |
| Glucose | mg/dl | 0.0555 | mmol/l |
| Insulin | μIU/ml | 6.945 | pmol/l |
| Glucagon | pg/ml | 1.0 | ng/l |
| C-peptide | ng/ml | 0.333 | nmol/l |
| Cholesterol | mg/dl | 0.0259 | mmol/l |
| Triglycerides | mg/dl | 0.0113 | mmol/l |

^a^To convert from the conventional unit to the SI unit, multiply by the conversion factor.

SI, Systeme International.
